# Whole tissue homogenization preferable to mucosal scraping in determining the temporal profile of segmented filamentous bacteria in the ileum of weanling rats

**DOI:** 10.1099/acmi.0.000218

**Published:** 2021-03-23

**Authors:** Linda A. Oemcke, Rachel C. Anderson, Jasna Rakonjac, Warren C. McNabb, Nicole C. Roy

**Affiliations:** ^1^​ Riddet Institute, Massey University, Palmerston North, New Zealand; ^2^​ School of Food and Advanced Technology, Massey University, Palmerston North, New Zealand; ^3^​ AgResearch, Grasslands Research Centre, Palmerston North, New Zealand; ^4^​ School of Fundamental Sciences, Massey University, Palmerston North, New Zealand; ^5^​ High-Value Nutrition National Science Challenge, Auckland, New Zealand; ^6^​ Department of Human Nutrition, University of Otago, Dunedin, New Zealand; ^7^​ Liggins Institute, University of Auckland, Auckland, New Zealand

**Keywords:** segmented filamentous bacteria, ileum, whole tissue homogenization, mucosal scraping, interleukin 17, immunoglobulin A

## Abstract

Segmented filamentous bacteria (SFB) are thought to play a role in small intestine immunological maturation. Studies in weanling mice have shown a positive correlation between ileal SFB abundance and plasma and faecal interleukin 17 (IL-17) and immunoglobulin A (IgA) concentrations. Although the first observation of SFB presence was reported in rats, most studies use mice. The size of the mouse ileum is a limitation whereas the rat could be a suitable alternative for sufficient samples. Changes in SFB abundance over time in rats were hypothesized to follow the pattern reported in mice and infants. We characterized the profile of SFB colonization in the ileum tissue and contents and its correlation with two immune markers of gastrointestinal tract (GIT) maturation. We also compared two published ileum collection techniques to determine which yields data on SFB abundance with least variability. Whole ileal tissue and ileal mucosal scrapings were collected from 20- to 32-day-old Sprague-Dawley rats. SFB abundance was quantified from proximal, middle and distal ileal tissues, contents and faeces by quantitative PCR using SFB-specific primers. Antibody-specific ELISAs were used to determine IL-17 and IgA concentrations. Significant differences in SFB abundance were observed from whole and scraped tissues peaking at day 22. Variability in whole ileum data was less, favouring it as a better collection technique. A similar pattern of SFB abundance was observed in ileum contents and faeces peaking at day 24, suggesting faeces can be a proxy for ileal SFB abundance. SFB abundance at day 26 was higher in females than males across all samples. There were significant differences in IgA concentration between days 20, 30 and 32 and none in IL-17 concentration, which was different from reports in mice and infants.

## Introduction

The identification of segmented filamentous bacteria (SFB) naturally occurring in invertebrates [1] and vertebrates [2–5] over 150 years ago [1] created an interest in these microbes. They are Gram-positive, spore-forming, facultative anaerobic commensals, which attach selectively to the mucosa of the terminal ileum [6–8] overlying Peyer’s patches, where naïve T cells undergo antigen-driven activation and expansion to yield T helper cells [9, 10]. The current literature reports that SFB mainly reside in the ileum [6, 11, 12] rather than the large intestine with the absence of Peyer’s patches in the large intestine [13] possibly contributing to this. Additionally, the selective colonization of the ileum mucosa by SFB might be explained by their ability to survive the microaerophilic conditions present in the ileum [14, 15]. The investigation into their effects during postnatal development has given insight into a symbiotic relationship with the host, primarily due to their suggested link with markers of immune maturation of the small intestine [16–20].

Commensal and pathogenic bacteria are known to modulate the abundance and activity of many immune markers of the maturation of the GIT. Both beneficial and detrimental effects on the host have been reported [21–24], and this is also the case for SFB. For instance, in adulthood, the abundance of SFB in stool samples has been positively associated with autoimmune and inflammatory bowel disease [11, 25, 26]. Studies have reported SFB to induce the production of the pro-inflammatory cytokine interleukin 17 (IL-17) in plasma [16–19, 27], and the antibody immunoglobulin A (IgA) in ileal contents and faeces of 4–6-week-old mice [20, 28, 29]. They also reportedly cause a weak inflammatory response in adult mice and children between 6 months and 15 years of age [16–19, 27]. The dampened response might be due to the lack of clostridial virulence-related genes [30] or absence of gene-encoding sortase, the enzyme that mediates anchoring of most cell-wall proteins in some pathogenic Gram-positive bacteria [31], from the SFB genome. Further to that, benefits of SFB have been suggested, including type 1 diabetes protection in adult non-obese diabetic mice involving IL-17 [32] and prevention of rotavirus infection in adult mice though independent of immune cell involvement [33].

The SFB were initially observed by microscope in the ileum tissue of rats [34], though most published studies on SFB have been performed in mice [2, 17, 18, 20, 30, 35–37]. It is unclear whether SFB in rodents are detectable prenatally up to 2 weeks postnatally. SFB appear about 3 weeks postnatally in mice and rats [20], increase at weaning [38], peak then plateau about 4 weeks later [20, 27, 39], highlighting their transient colonization profile. SFB may, therefore, provide benefits primarily around weaning, likely preventing overstimulation of the immune system later in life.

There are also technical limitations in the quantification of SFB. *In vitro* studies have been challenging as these microbes thrive in environments of low to no oxygen concentrations while requiring an attachment to epithelial cells that need oxygen [14, 40]. Human infant studies rely on faecal samples as a proxy of ileal abundance, as there are ethical challenges associated with obtaining ileal samples. Mice have been the most frequently used animal model to study SFB pre- and post-weaning, though this is not without challenges; for example, obtaining enough ileal tissue and content samples for accurate SFB quantification and conducting multiple analyses to characterise its effects on the host.

Two methods for obtaining ileal samples for SFB quantification have been described in the published literature [2, 20, 41]. Ericsson *et al*. [2] attempted to create pure inocula of SFB by developing a cost-effective method of isolating SFB from complex microbes in the ileum. Using an aseptic technique, they rinsed the distal ileum of 2–3-day-old SFB-positive Balb/c mice. The ileal tissue was cut longitudinally exposing the mucosa, which was then scraped with a sterile scalpel blade. The scrapes contained epithelial cells, and mucosa-associated bacteria (including SFB) were then transferred into sterile media. Ohashi *et al*. [20] used a grinding method [41] to collect ileum tissue along with mucosa-associated bacteria. This method involves grinding whole tissue with a pestle and mortar on ice then transferring the ground up tissue into a buffer for DNA extraction.

Therefore, the study hypothesis was that changes in SFB abundance in ileal tissue and contents over time in rats would have a similar pattern to that reported in mice and infants. A weanling rat model was used to evaluate the profile of SFB colonization and some markers of immune maturation of the ileum pre- and post-weaning. Two techniques used in published studies for collecting ileum tissue samples for SFB quantification, ileal mucosal scraping [2] and whole tissue grinding or homogenization [41], were compared to determine whether one technique would yield better results with reduced variability. Following the use of these two techniques, the abundance of SFB was quantified by quantitative PCR (qPCR), and IL-17 and IgA concentrations by ELISA.

## Methods

### Rat experiment

The study was approved by the AgResearch Limited Grasslands Animal Ethics Committee (Animal Ethics Application No: 14485) under the recommendations of the New Zealand Animal Welfare Act 1999.

Conventionally raised Sprague-Dawley rat pups (male and female) were used. Eight pregnant Sprague-Dawley dams were obtained from AgResearch Ruakura (Hamilton, NZ) and transported to AgResearch Grasslands (Palmerston North, NZ) at 15 days of gestation. They were maintained at 21 °C and provided with a commercial rodent diet (Meat Free Rat and Mouse Diet, Specialty Feeds, Australia) and water *ad libitum*. The dams were individually caged under dark and light cycles. Five litters were birthed on the first day, two litters on the second day and one litter on the third day. All dams and pups were maintained at 21 °C. The pups were weaned on day 21, and the dams were removed from the birth cages.

Ninety-two pups (57 females and 35 males) were evenly distributed across time-points. The pups were randomized into seven time-points: 20, 22, 24, 26, 28, 30 and 32 days after birth (*n*=13 for day 20, *n*=14 for others). They were identified by ear-punching at day 19 to meet the welfare norms for this procedure. The pups remained in their birth litters throughout the experiment to avoid interaction among animals from different litters which would influence the gut microbiota of the pups. All dams and pups were checked daily, weighed once a week, and fresh food and water topped up as required. Their General Health Score (GHS), which ranges from 5 (healthy) to 1 (requires euthanization) was checked daily, and the rats with a GHS of 3 or 4 were closely monitored. If their condition deteriorated, they were euthanized by intraperitoneal injection with pentobarbitol. All rats had a GHS of 5 throughout the study.

### Sample collection

**Fig. 1.**
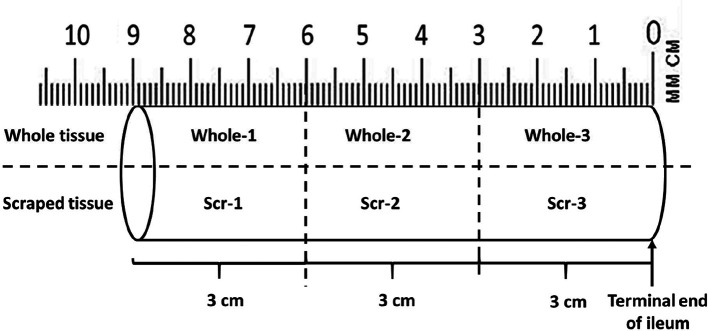
Schematic of how the ileum tissue was cut into three sections of 3 cm each. The ileum sections were measured up from the caecum, which borders the terminal end of the ileum. The sections were cut into 3 cm each, cut open and down the middle longitudinally. The top sections were collected as whole tissue samples. The bottom sections were scraped and used. Both whole and scraped tissue were homogenized before genomic DNA extraction. 1-proximal section, 2-middle section, 3-terminal section.

At each sampling time point, the pups were euthanized by asphyxiation with carbon dioxide in an individual cage followed by cervical dislocation. Blood was drawn by cardiac puncture into a needle coated with Ethylenediaminetetraacetic acid (EDTA) (Invitrogen; Thermo Fisher Scientific). The blood samples were centrifuged at 2000 ***g*** for 10 min at 4 °C, and the supernatant (plasma) was pipetted into cryotubes and snap-frozen in liquid nitrogen before storage at −80 °C for later analysis. Faecal samples were collected from the terminal colon or rectum post-mortem and snap-frozen in liquid nitrogen and stored at −80 °C for DNA extraction. The distal portion of the ileum adjacent to the caecum was cut into three sections of 3 cm each. The ileal contents from each section were collected separately. The ileal tissue sections were then cut open longitudinally and down the middle into two pieces. The top sections of the tissue were collected as whole tissue. The bottom sections were each scraped three times with a plastic tissue scraper in the same direction using a similar amount of force to decrease variability in the sample weights (Fig. 1). The scrapings and whole tissues were collected into cryotubes and snap-frozen in liquid nitrogen and stored at −80 °C for DNA extraction.

### Sample preparation and genomic DNA extraction

Genomic DNA was extracted from the whole and scraped ileum tissue samples using the Qiagen AllPrep DNA/RNA/Protein Mini Kit according to the manufacturer’s instructions. Before DNA extraction, both tissue samples were disrupted using a tissue homogeniser (Omni International TH, Georgia, USA). The homogenizer probe was cleaned with two different solutions of 70 % ethanol, 100 % ethanol and distilled water between each sample to prevent cross-contamination. DNA was extracted from ileal contents and faeces using the NucleoSpin Soil extraction kit (Macherey-Nagel) according to the manufacturer’s instructions. These samples were vigorously agitated in a bead beater to release the DNA from the SFB intracellular contents and spores. The concentration and purity of DNA from ileum tissue, ileum contents and faeces were measured using a Nanodrop ND-1000 Spectrophotometer (Analytical Technologies, Thermo Fisher Scientific, MA, USA). A ratio of 1.8 was considered acceptable for pure DNA. The DNA samples were then ready for quantification by qPCR.

### QuantitativePCR to quantify SFB

An optimized protocol based on the one from the Franklin Laboratory in St. Louis, Missouri, USA (personal communication) was utilized for the qPCR analysis. A standard curve was generated using a recombinant plasmid DNA containing the SFB 16S rRNA gene fragment (Integrated DNA technologies). The master mix consisted of 5 µl of KAPA SYBR Green I, 0.2 µl of Rox low reference dye (Sigma-Aldrich), 0.2 µl each of forward and reverse primers (Integrated DNA Technologies), and 3.4 µl of nuclease-free water (Invitrogen). To this mix, 1 µl of the recombinant plasmid DNA (positive control), 1 µl of nuclease-free water (negative control) or 1 µl of sample DNA (ileum tissue, contents, or faecal) were added for a total of 10 µl in each reaction well on a 96-well plate. The technical replicates used were four each. The SFB primers were forward primer SFB 779F 5′- TGT GGG TTG TGA ATA ACA AT −3′, reverse primer SFB 1008R 5′- GCG AGC TTC CCT CAT TAC AAG G −3′ [42] (Integrated DNA technologies). The conditions in the Quantstudio 3D Digital PCR thermal cycler (Applied Biosystems; Thermo Fisher Scientific, MA, USA) included a hold stage at 95 °C for 3 min, the PCR Stage; 40 cycles of denaturation at 95 °C for 3 min; annealing at 64 °C for 30 s; extension at 72 °C for 30 s. A melting-curve analysis using SYBR green was performed to determine the specificity of the PCR by slowly heating the mixtures from 55–95 °C for 1 s, 60 °C for 30 s and 95 °C for 1 s. Duplicate negative controls were run to assess the specificity and to rule out contamination. Data were analysed, and the relative quantification (fold) of SFB DNA was performed using the ∆∆Ct method.

### ELISA of immune markers

The concentrations of IL-17 in plasma and IgA in faeces from male and female rats were analysed using an antibody-specific Rat IL-17 ELISA Kit and Rat IgA ELISA Kit (Cusabio Biotech) according to the manufacturer’s instructions. A FlexStation 3 Multi-Mode Microplate Reader (Molecular Devices, CA, USA) was used to determine the optical density (OD). The OD was measured at 450 nm, which was subtracted from 540 nm to determine the final OD readings. Standard curves, used to change the raw data OD readings to concentrations, were created for IL-17 and IgA using Genstat (18th Edition). Linear and polynomial regressions were generated by plotting the mean absorbance for each standard against the concentration of plasma IL-17 and faecal IgA, respectively. The limit of detection for plasma IL-17 and faecal IgA was 0.05 ng ml^−1^ and 0.88 ng ml^−1^, respectively.

### Statistical analysis

A one-way ANOVA was performed in Minitab 18 to determine if there were any significant differences in the abundance of SFB and concentration of IL-17 and IgA over time between days 20–32 pre- and post-weaning. A two-way ANOVA was also performed to determine whether the interaction between sex and age influenced SFB abundance over time. The Ryan-Joiner’s (like Shapiro–Wilk) test and Levene’s test were used to verify that the data were normally distributed, and that homogeneity of variance was met. The data were log-transformed to meet the requirements of normal distribution and homogeneity of variance. The one-way ANOVA was performed using the logarithm-transformed data. Where there was statistical significance, a post-hoc Tukey’s test was performed to show where the differences lay. Differences were considered statistically significant when the probability value was inferior to 0.05. An analysis of covariance (ANCOVA) was performed to determine whether the sex of the rats influenced their weight gain with minimal effects from age. Weight gain was the response, sex was the explanatory factor, and age was the covariate (control variable).

## Results

### Quantification of SFB

**Fig. 2.**
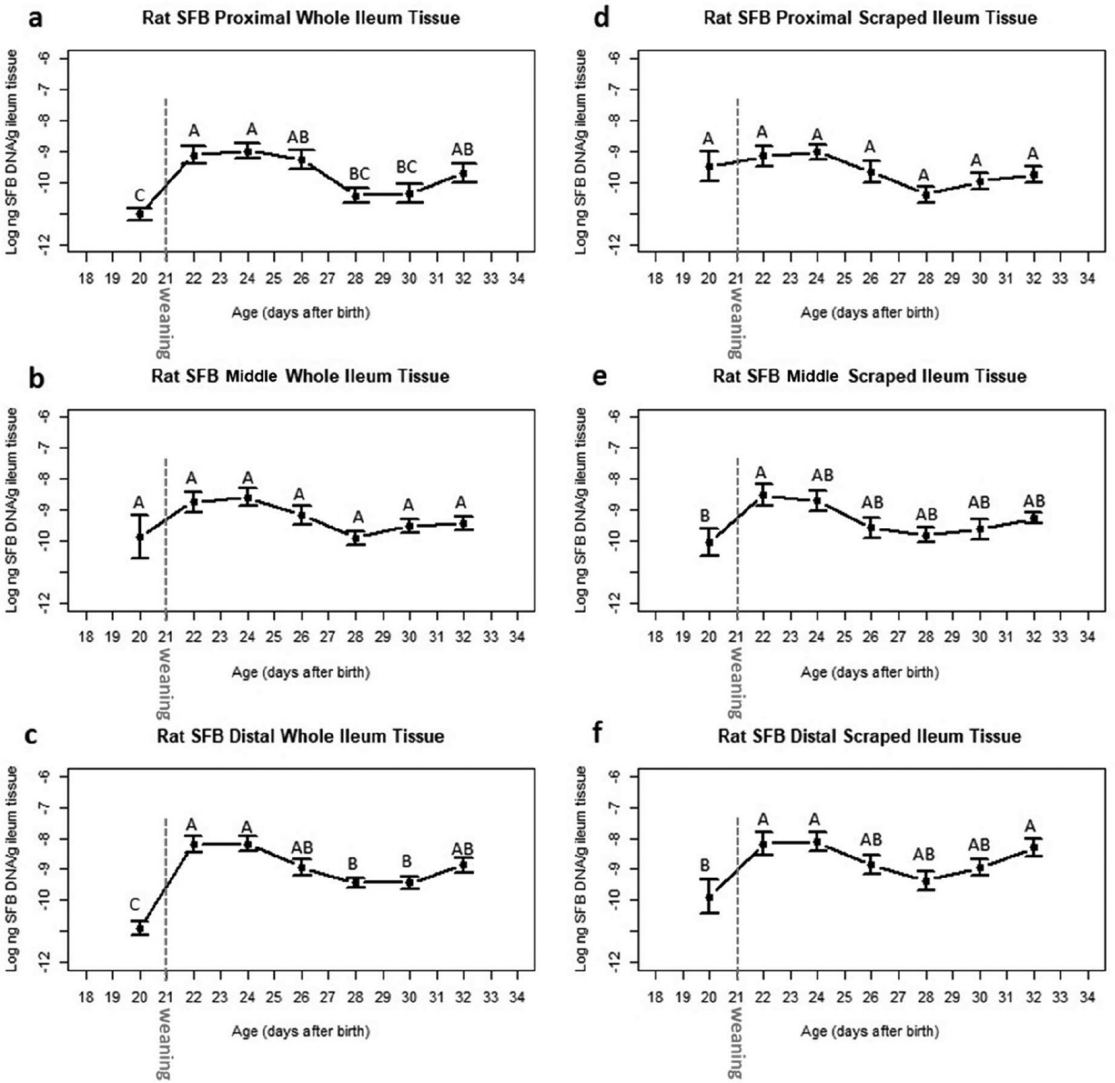
Abundance of SFB in the whole ileal tissue (a–c) and ileal mucosal scrapes (d–f) of conventionally reared Sprague-Dawley rat pups. Samples were collected from the proximal, middle and distal ileum. Data are plotted on a logarithm ten scale and are shown as the mean values of *n*=14 pups (20 days postnatally) and *n*=13 pups (22–32 days postnatally). The bars represent the sem. Values without common letters differ significantly *P* <0.05.

**Fig. 3.**
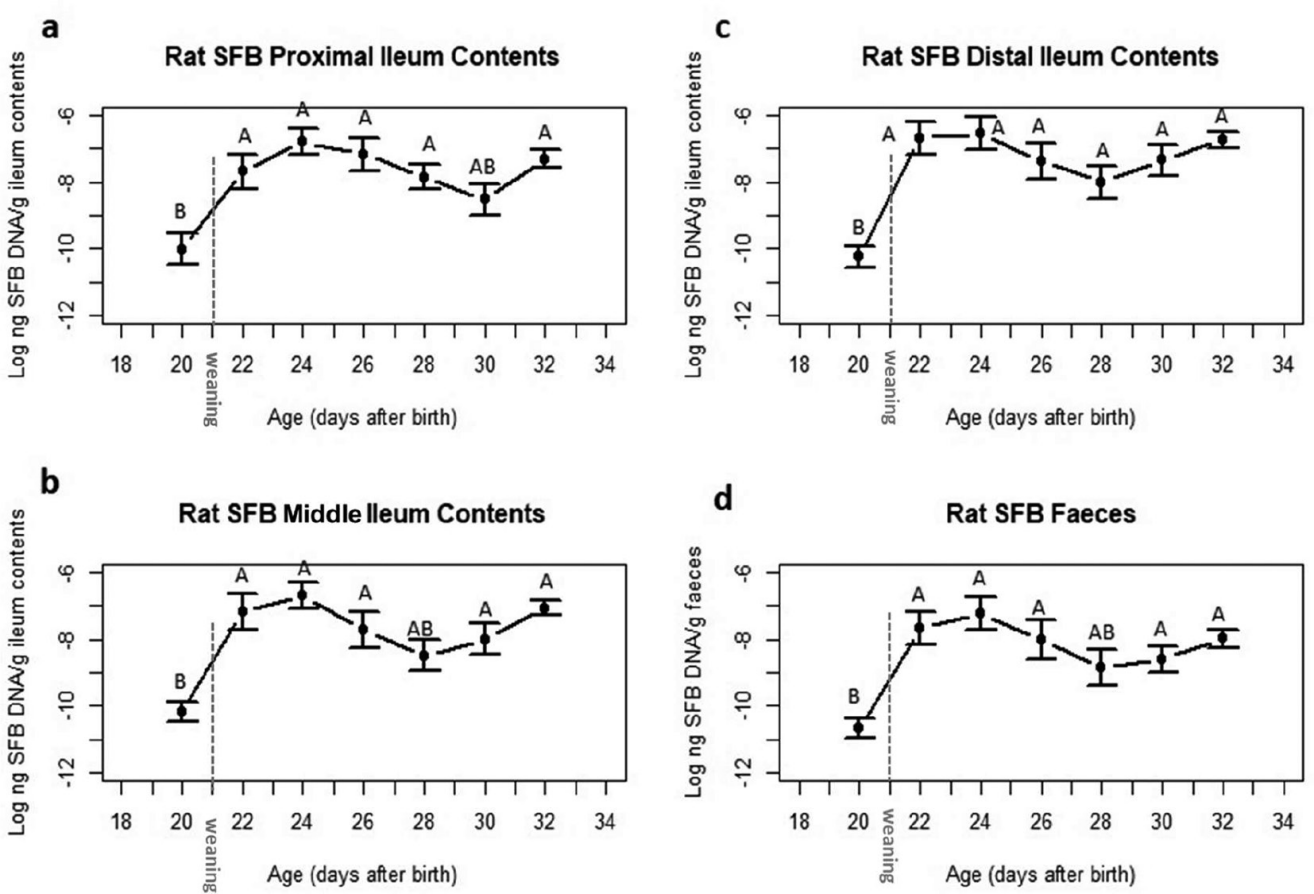
The abundance of SFB in the ileal content (a–c) and faecal (d) samples collected from conventionally reared Sprague-Dawley rat pups. Samples were collected from the proximal, middle and distal ileum. Data are plotted on a logarithm ten scale and are shown as the mean values of *n*=14 pups (20 days postnatally) and *n*=13 pups (22–32 days postnatally). The bars represent the sem. Values without common letters differ significantly *P* <0.05.

The initial data of the abundance of SFB were skewed, therefore, to meet conditions of normality the data were transformed and plotted on a logarithm ten scale. All figures with untransformed data are included in the Supplementary Material, available in the online version of this article. The abundance of SFB increased from weaning, peaked at PND 22, and then plateaued (Figs 2a–f and S1a–f). Significant differences were observed in the temporal abundance of SFB in the proximal (Figs 2a and S1a) and distal (Figs 2c and S1c) sections of the whole ileum tissue from day 20, peaking at day 22 then decreasing from day 26, but not in the middle (Figs 2b and S1b) section. For the scraped ileum tissue, significant differences were observed in the temporal abundance of SFB in the middle (Figs 2e and S1e) and distal (Figs 2f and S1f) sections from day 20, peaking at day 22 then decreasing from day 26, but not in the proximal (Figs 2d and S1d) section. In all cases, SFB abundance peaked at day 22 and then plateaued. There was less variability in SFB abundance in the proximal, middle and distal whole ileum tissue (Fig. 2a–c; average sem *: 2.71E-01;* Fig. S1a–c; average sem: *1.84E-07*) compared to that from the proximal, middle, and distal ileal scrapes (Fig. 2d–f; average sem: *3.18E-01;* Fig. S1d–f; average sem: *6.10E-07*). There were significant differences in the abundance of SFB in the proximal (Figs 3a and S2a) and middle (Figs 3b and S2b) section of ileal contents over time between postnatal days 20 and postnatal days 22, 24, 26, 28 and 32. There were no significant differences in SFB abundance in ileal contents from the distal (Figs 3c and S2c) section between day 22 and 32.

There were significant differences in SFB abundance over time in the faeces (Figs 3d and S2d) increasing from day 20, peaking at day 24, then decreasing from day 28. The faecal SFB abundance peaked later at day 24 compared to the ileum tissue at day 22 though there were no significant differences.

**Fig. 4.**
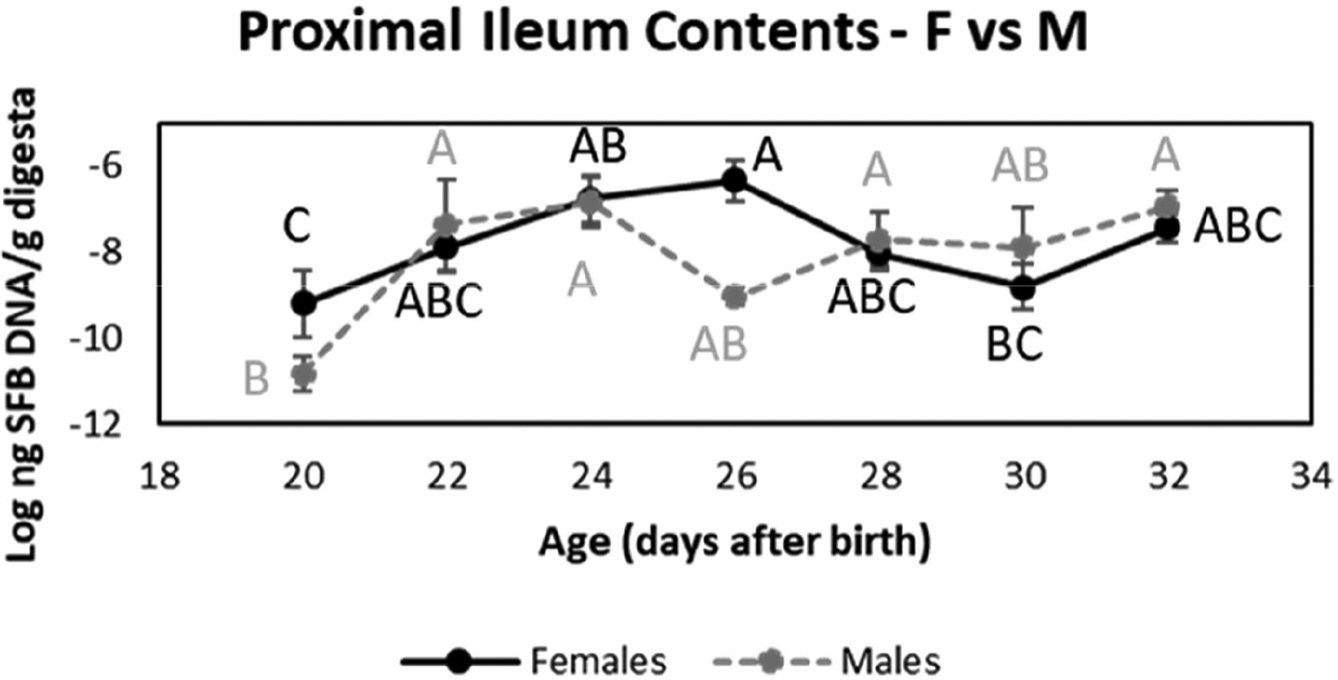
Comparison of the abundance of SFB in the proximal ileal content samples between female and male Sprague-Dawley rat pups. The rat pups were weaned on day 21. Data are plotted on a logarithm ten scale and are shown as the mean values of females (*n*=57) versus males (*n*=35). The bars represent the sem. Values without common letters differ significantly *P*<0.05.

There were significant differences in the interaction between sex and age on SFB abundance in the proximal ileum contents only. There was an unexpected difference in SFB abundance at day 26 between male and female pups. SFB abundance in males sharply decreased while in females, there was a gradual decrease until the plateau was reached at day 28 (Figs 4 and S3). The male pups also gained weight quicker than females (Table 1). There were significant differences in weight gain between males and females when age was used as a covariate.

**Table 1.** Mean±sem wt gain in grams (g) of pre- and post-weaning male and female Sprague-Dawley rats. The values represent weight gained between the initial weigh-ins (at postnatal days 15, 17, 18) and before sample collection from pups at all time-points. Compared to females, males gained more weight, and male pups were the heaviest at terminal sample collection. An ANOVA and post-hoc Tukey’s test showed there were significant differences in average weight gain in males among time-points 1–2, 4–6 and 7. There were significant differences in average weight gain in females at all time-points; *P* <0.05

Time-point	Males	Females
1	7.43±0.98^d^	7.00 ± 0.61^g^
2	16.92 ± 1.74^cd^	15.76 ± 1.08^f^
3	25.80 ± 2.61^c^	26.74±1.60^e^
4	44.48±2.41^b^	39.44±1.08^d^
5	56.20±3.56^b^	50.85 ± 2.86^c^
6	53.23±4.54^b^	60.27±2.58^b^
7	92.93±11.52^a^	74.56±1.72^a^

### Analysis of IgA and IL-17

**Fig. 5.**
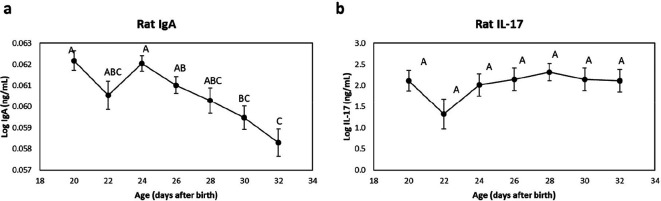
Concentration of IgA (a) in faeces and IL-17 (b) in plasma of conventionally reared male and female Sprague-Dawley rat pups. The limit of detection for faecal IgA was 0.88 ng ml^−1^, and plasma IL-17 was 0.05 ng ml^−1^, respectively. Data are plotted on a *logarithm ten* s*c*ale and are shown as the mean values of *n*=13 pups (22–32 days) and *n*=14 pups (20 days). The bars represent the sem. Values without common letters differ significantly *P* <0.05.

The IgA and IL-17 data were transformed and plotted on a logarithm ten scale to meet conditions of normality. All figures with untransformed data are included in the Supplementary Material. There were significant differences in the concentration of IgA in faeces (Figs 5a and S4a), which decreased postnatally from day 24 to 32. Tukey’s post-hoc test showed that there were differences in the faecal IgA concentration between days 20, 24 and days 30, 32. There were no significant changes in the concentration of IL-17 in plasma over time (Figs 5b and S4b).

## Discussion

This study shows that the abundance of SFB in ileum tissue and content of rats measured pre- and post-weaning was similar to the temporal profiles published in mice and infants of corresponding age [20, 39]. Here, the abundance of SFB increased post-weaning, peaked at days 22 and 24 and decreased until a plateau was reached at day 26 until the last measurement on day 32. Other studies suggest that the abundance of SFB remains relatively constant after weaning throughout life [27, 39].

The results also showed that of the two tissue collection techniques, whole tissue homogenization gave less variable tissue weights compared with mucosal scraping. SFB abundance data was similar to what has been reported [20]. Interestingly, the SFB data from homogenization was less variable compared to the scraped data. High variability in the scraped data was likely partly due to scraped tissue sticking to the collection tools, thus decreasing the amount of tissue available. The fragility of the ileum at that age also made scraping of the tissue challenging, resulting in variable weights. Whole tissue homogenisation was, therefore, a simpler method of collecting ileum tissue samples from rats for analysis pre- and post-weaning.

The pattern of SFB abundance in the ileum tissue and contents overtime was also reflected in the faeces, suggesting faeces could be used as a proxy for SFB abundance in the ileum. The faecal SFB abundance peaked later at day 24 compared to the ileum tissue at day 22; abundance in the ileum would, therefore, be assumed to peak earlier. This non-invasive approach of using faeces aligns with the three Rs of animal research that include replacement, reduction and refinement, which utilize alternatives to terminal animal sampling, reduce the number of animals used and follow procedures that minimize pain and stress [43]. This alternative to terminal sampling to obtain ileal tissue content could be applied in time-series and intervention studies attempting to understand influences on SFB abundance caused by diet, disease, medication or ageing [11].

The significant difference in the interaction of sex with age indicates that SFB abundance in the proximal ileum contents over time is different in males and females. It suggests that this interaction may be present in the middle and distal ileum contents though a study with more statistical power would be required to make this conclusion. The abundance of SFB was unexpectedly different between females and males on day 26 only. This result may be a random difference in the rats sampled on that day. The observation also suggests that sex-based hormonal differences might influence SFB abundance. It may result from physiological differences in GIT development between sexes [44] as the male pups gained weight quicker. Before puberty, oestradiol concentrations peak at day 15 in both sexes and remain low until day 39, while testosterone concentrations in both sexes remain low between days 1 and 19 then decrease between days 20 and 30 [45, 46]. Rats reach puberty between days 30 and 42 in females and days 42 and 55 in males [47]. Sex hormones were not measured here, so it is unclear whether a change in their concentrations leading up to puberty might have influenced SFB abundance in the samples analysed. The abundance of SFB between sexes was not reported in a similar study, which involved six male and nine female mouse pups [20]. Thus far, our study is the only one that has reported this finding, which highlights the importance of considering gut microbiota changes between sexes.

In this study, there was no difference in the plasma concentration of IL-17 from days 22 to 32. However, our previous (unpublished) results in mice showed differences in plasma concentration of IL-17 between days 18 and 41; the concentration peaked at day 32. These results also contrast with literature reports of a positive correlation between ileal SFB abundance and plasma IL-17 production in adult mice 2 to 3 weeks following SFB colonization [4, 16, 17]. Several plasma samples across all time-points measured here had IL-17 concentrations lower than the calculated detection threshold, increasing the variability in the data. At day 22, most of the rats had zero-readings, and no measurements were done after day 32.

The faecal concentration of IgA was variable from days 20 to 28 then decreased until day 32. This result is in disagreement with other studies where faecal IgA concentrations reportedly increased only late post-weaning [20, 48]. Other studies showed that oral inoculation of SFB in older conventional and germ-free mice initially stimulates the production of IgA [48], with IgA later inhibiting SFB colonization [20, 49]. It is plausible that breastmilk IgA may have contributed to the high IgA concentrations found in the faecal samples at day 20, as observed in caecal contents of pre-weaned mice [20]. Other microbes may be involved, as they change in abundance around weaning, and are also known to stimulate production and secretion of IgA by immune cells [21, 23, 29]. The numerical drop of faecal IgA concentration at day 22 may have resulted from weaning on day 21, then the increase at day 24 may have resulted from increased luminal IgA. A broader age range, with a later developmental stage, may give a clearer picture of how IgA concentration changes [20] as the duration of this study was short.

In conclusion, the results showed that the temporal profile of SFB colonization in the ileum tissue and content of weanling rats was similar to those published for mice and infants of corresponding age. Lower variability in whole ileal tissue data favours it as a preferred tissue collection technique. The data also show that faeces can also be used as a proxy for SFB abundance in the ileum. The immune markers did not give similar results to those reported in mice and infants making it difficult to conclude on the influence of SFB on IL-17 and IgA. This indicates the importance of investigations on the temporal profile of SFB and their effects on IL-17 and IgA, particularly at weaning. The results may determine whether the studies are reproducible and contribute to current knowledge of SFB. The study model and results may also help guide the design of further studies investigating the perceived role of SFB on small intestine immunological barrier maturation.

## Supplementary Data

Supplementary material 1
