# Supplementary material for: Whole tissue homogenization preferable to mucosal scraping in determining the temporal profile of segmented filamentous bacteria in the ileum of weanling rats
Source: Access Microbiol. 2021 Mar 23;3(3):000218. doi: 10.1099/acmi.0.000218 (PMC8209713; doi:10.1099/acmi.0.000218)
Supplement: Supplementary material 1 [file acmi-3-0218-s001.pdf]

Supplementary Figures

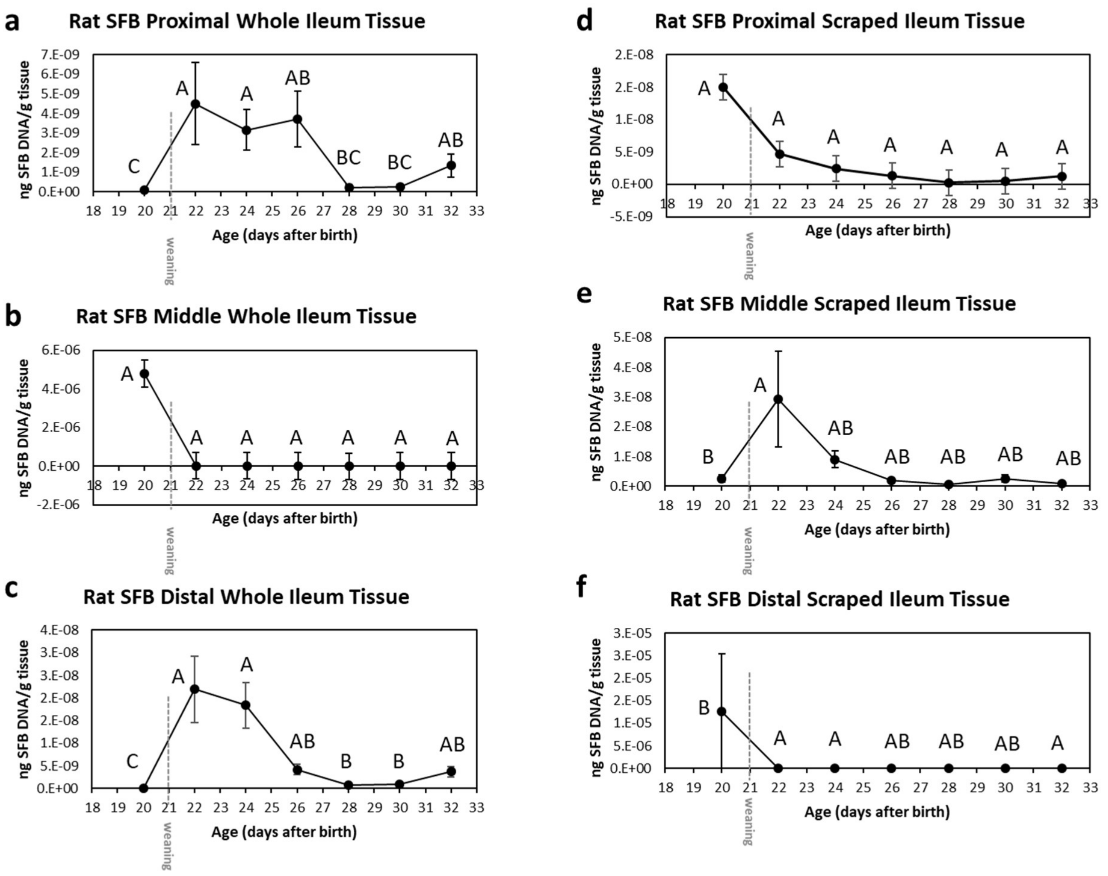

Figure 1. Abundance of SFB in the whole ileal tissue (a, b, c) and ileal mucosal scrapes (d, e, f) of conventionally reared Sprague-Dawley rat pups. Samples were collected from the proximal, middle and distal ileum. Data are untransformed and are shown as the mean values of n=14 pups (20 days postnatally) and n=13 pups (22-32 days postnatally). The bars represent the standard error mean (SEM). Values without common letters differ significantly $p < 0.05$.

Company Limited by Guarantee l Registered in England No. 1039582 l Registered Office Charles Darwin House, 12 Roger Street, WC1N 2JU, UK
Registered as a Charity: 264017 (England & Wales); SC039250 (Scotland)

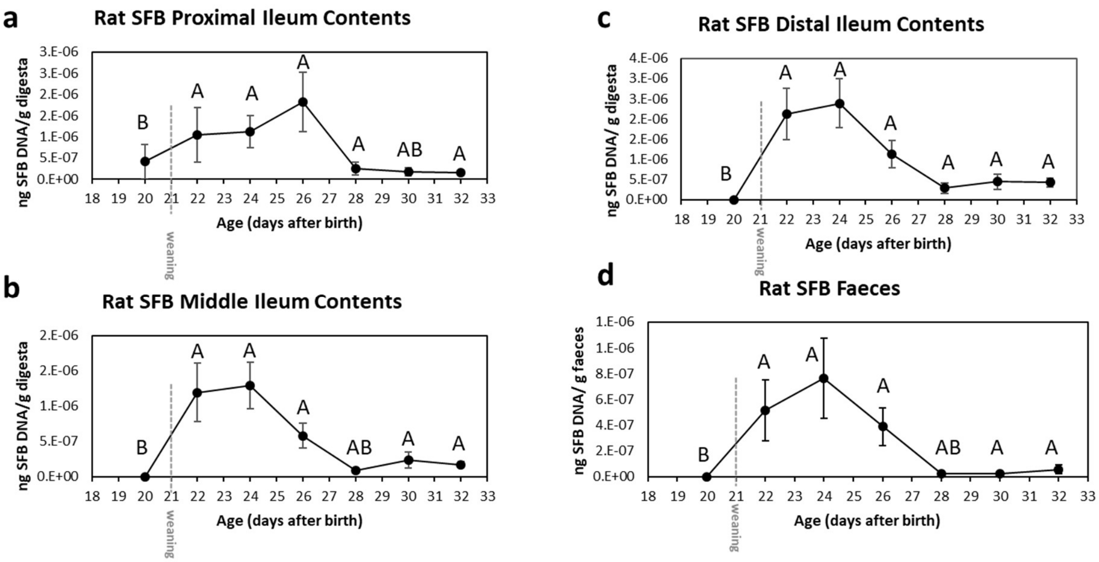

Figure 2. The abundance of SFB in the ileal content (a, b, c) and faecal (d) samples collected from conventionally reared Sprague-Dawley rat pups. Samples were collected from the proximal, middle and distal ileum. Data are untransformed and are shown as the mean values of n=14 pups (20 days postnatally) and n=13 pups (22-32 days postnatally). The bars represent the standard error mean (SEM). Values without common letters differ significantly $p < 0.05$.

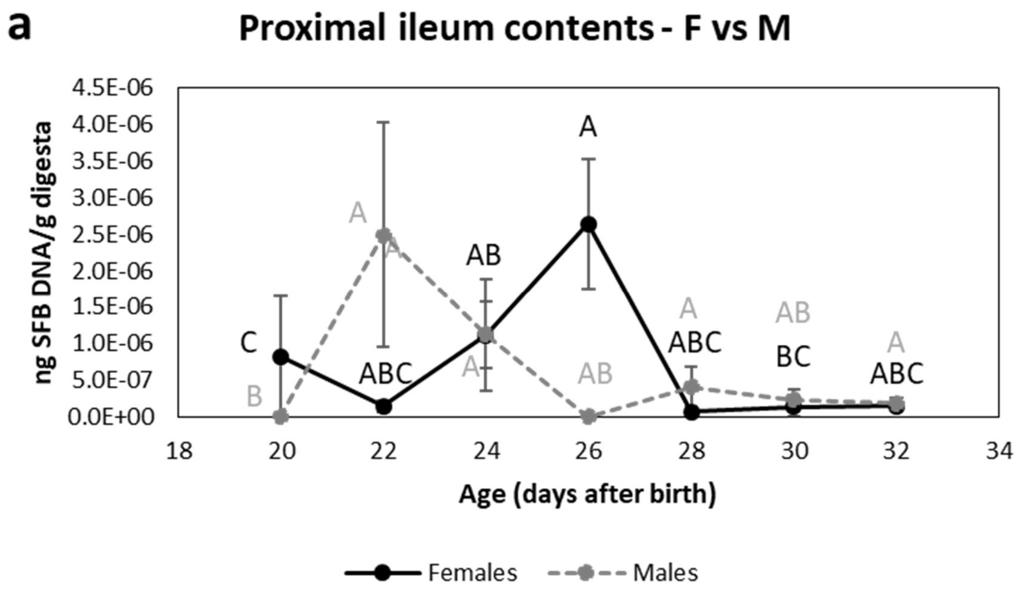

Figure 3. Comparison of the abundance of SFB in the proximal ileal content samples between female and male Sprague-Dawley rat pups. Data are untransformed and are shown as the mean values of females (n=57) versus males (n=35). The bars represent the standard error mean (SEM). Values without common letters differ significantly $p < 0.05$.

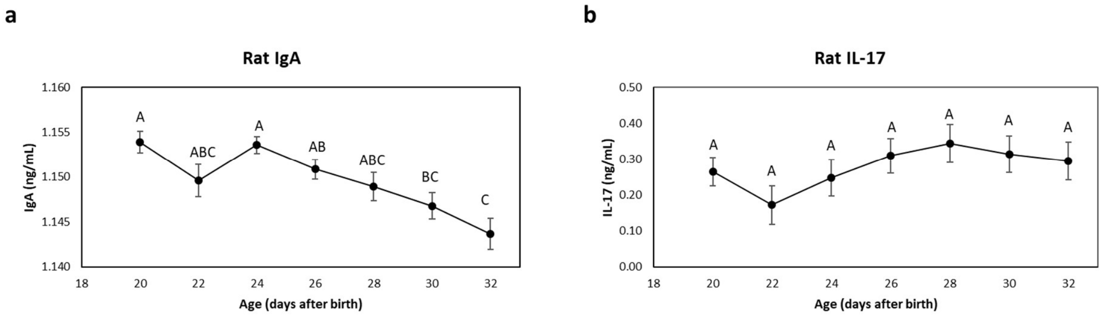

Figure 4. Concentration of IgA (a) in faeces and IL-17 (b) in plasma of conventionally reared male and female Sprague-Dawley rat pups. The limit of detection for faecal IgA was 0.88 ng/mL, and plasma IL-17 was 0.05 ng/mL, respectively. Data are untransformed and are shown as the mean values of n=13 pups (22-32 days) and n=14 pups (20 days). The bars represent the standard error mean (SEM). Values without common letters differ significantly $p < 0.05$.
